# Co-continuous network polymers using epoxy monolith for the design of tough materials

**DOI:** 10.1038/s41598-021-80978-2

**Published:** 2021-01-14

**Authors:** Ren Tominaga, Yukihiro Nishimura, Yasuhito Suzuki, Yoshihiro Takeda, Masaru Kotera, Akikazu Matsumoto

**Affiliations:** 1 Department of Applied Chemistry, Graduate School of Engineering, Osaka Prefecture University, 1-1, Gakuen-cho, Naka-ku, Sakai, Osaka 599-8531 Japan; 2 Core Technology Research Department, X-Ray Research Laboratory, Rigaku Corporation, 3-9-12, Matsubara-cho, Akishima, Tokyo 196-8666 Japan; 3 R&D Department, Hotmelt Adhesive Division, MORESCO Corporation, 5-5-3, Minatojimaminami-machi, Chuo-ku, Kobe, Hyogo 650-0047 Japan

**Keywords:** Chemistry, Materials science

## Abstract

High-performance polymer materials that can exhibit distinguished mechanical properties have been developed based on material design considering energy dissipation by sacrificial bond dissociation. We now propose co-continuous network polymers (CNPs) for the design of tough polymer materials. CNP is a new composite material fabricated by filling the three-dimensionally continuous pores of a hard epoxy monolith with any cross-linked polymer having a low glass transition temperature (*T*_g_). The structure and mechanical properties of the CNPs containing epoxy resins, thiol-ene thermosets, and polyacrylates as the low-*T*_g_ components were investigated by differential scanning calorimetry, dynamic mechanical analysis, tensile tests as well as scanning electron microscopic observations and non-destructive 3D X-ray imaging in order to clarify a mechanism for exhibiting an excellent strength and toughness. It has been demonstrated that the mechanical properties and fractural behavior of the CNPs significantly depend on the network structure of the filler polymers, and that a simultaneous high strength and toughness are achieved via the sacrificial fracture mechanism of epoxy-based hard materials with co-continuous network structures.

## Introduction

The toughening of polymer materials is the key for the design of high-performance materials used for our future society because it is indispensable not only for new material development, but also for solving energy problems in many fields^1–5^. Materials that can exhibit a high extension, toughness, and self-healing property have been developed based on some innovative ideas to provide flexibility and toughness to a polymer at the same time; e.g., double network (DN) gels^6^, slide-ring gels^7^, tetra-PEG gels^8^, organic‒inorganic nanohybrids^9–11^, dynamic covalent bond network polymers^12,13^, etc. Cross-linked polymers have an excellent heat resistance and strength, but, on the other hand, the network structure suppresses the sliding motion of the molecular chains as well as plastic deformation. The difficulty of absorption and dissipation of a high energy applied from the outside causes brittle fracture of the cross-linked materials. Therefore, reactive liquid rubber/elastomers^14,15^ or thermoplastic resins^16^ are used as the modifier to toughen hard materials with a network polymer structure. A toughening mechanism accompanying the cavitation of an elastomer phase and the plastic deformation of the matrix has been proposed based on the analysis of crack development in the materials^17–21^. It has been clarified that the filler concentration, size, and shape as well as interfacial adhesion significantly affect the toughness of various composite materials^1,2^, but it is still difficult to simultaneously achieve both the strength and flexibility for epoxy-based composites.

Hydrogels with various functions are used in a wide range of fields from biological and medical research to practical superabsorbent polymers and membrane separation processes^22,23^. Conventional hydrogels cannot escape from the characteristics of being soft and brittle, but DN gels with a structure, in which two types of networks are entangled with each other, have been developed as a new type of high-strength materials^6–8,24^. The concept of sacrificial bonding was applied to the high-strength DN gels, being first proposed as a mechanism for strengthening biological tissues^25^. The intelligent polymer materials exhibiting further advanced properties have continuously been developed by Gong et al. and other researchers^26^. The application of material toughening by a sacrificial fracture mechanism is not limited to interaction at the molecular level, but the toughening is also reported for composite materials with structural control on a large scale of millimeters or more^27^. In addition, Creton et al. demonstrated that a similar fracture mechanism could be applied to elastomeric rubber materials other than hydrogels^28^.

A monolith with a three-dimensional continuous network skeleton and through holes has been applied as a high-performance material, of which a high porosity and strength are used for the separation, reaction, and other functions^29,30^, e.g., HPLC columns^31,32^, supports for catalysts^33^, separators for lithium-ion batteries^34^, and dissimilar materials bonding^35–37^. We now propose a new strategy for the synthesis of toughened polymer materials using an epoxy monolith. In this study, we have successfully controlled the mechanical properties of co-continuous network polymers (CNPs) synthesized by filling the continuous pores of an epoxy monolith with different cured resins. We fabricated three kinds of CNPs containing epoxy resins, thiol‒ene thermosets, and polyacrylates as the cross-linked second polymers in order to control the strength and toughness by a sacrificial fracture mechanism. The structure, thermal, and mechanical properties of the monolith and the fabricated CNPs were investigated.

## Epoxy monolith

**Figure 1:**
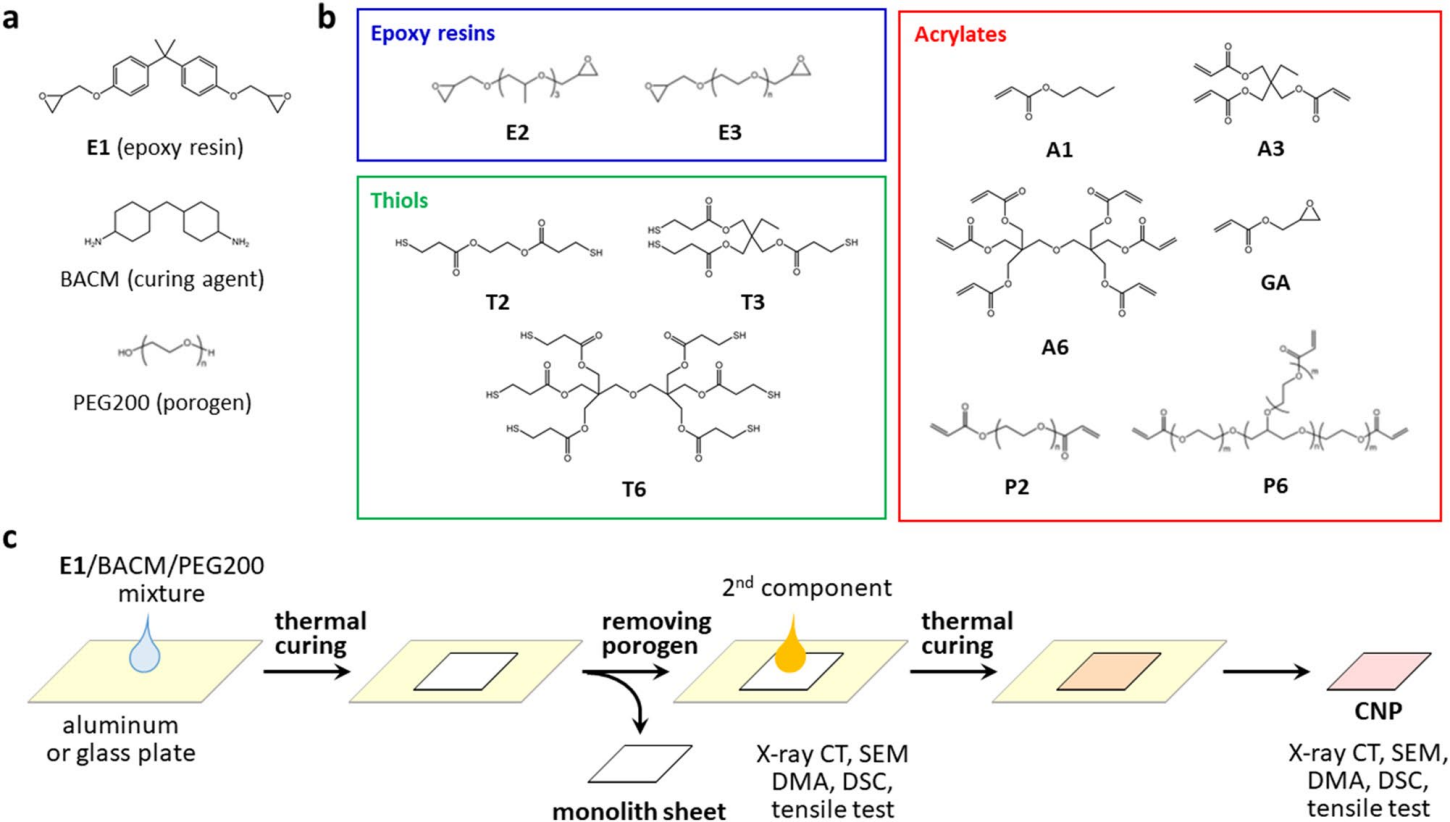
Process for the fabrication of epoxy monolith and CNP. (**a**) Chemical structure of compounds used for the fabrication of epoxy monolith. (**b**) Chemical structure of compounds used as the second component of the CNPs. **c,** Schematic illustration for the fabrication and characterization of epoxy monolith sheets and the CNPs.

**Figure 2:**
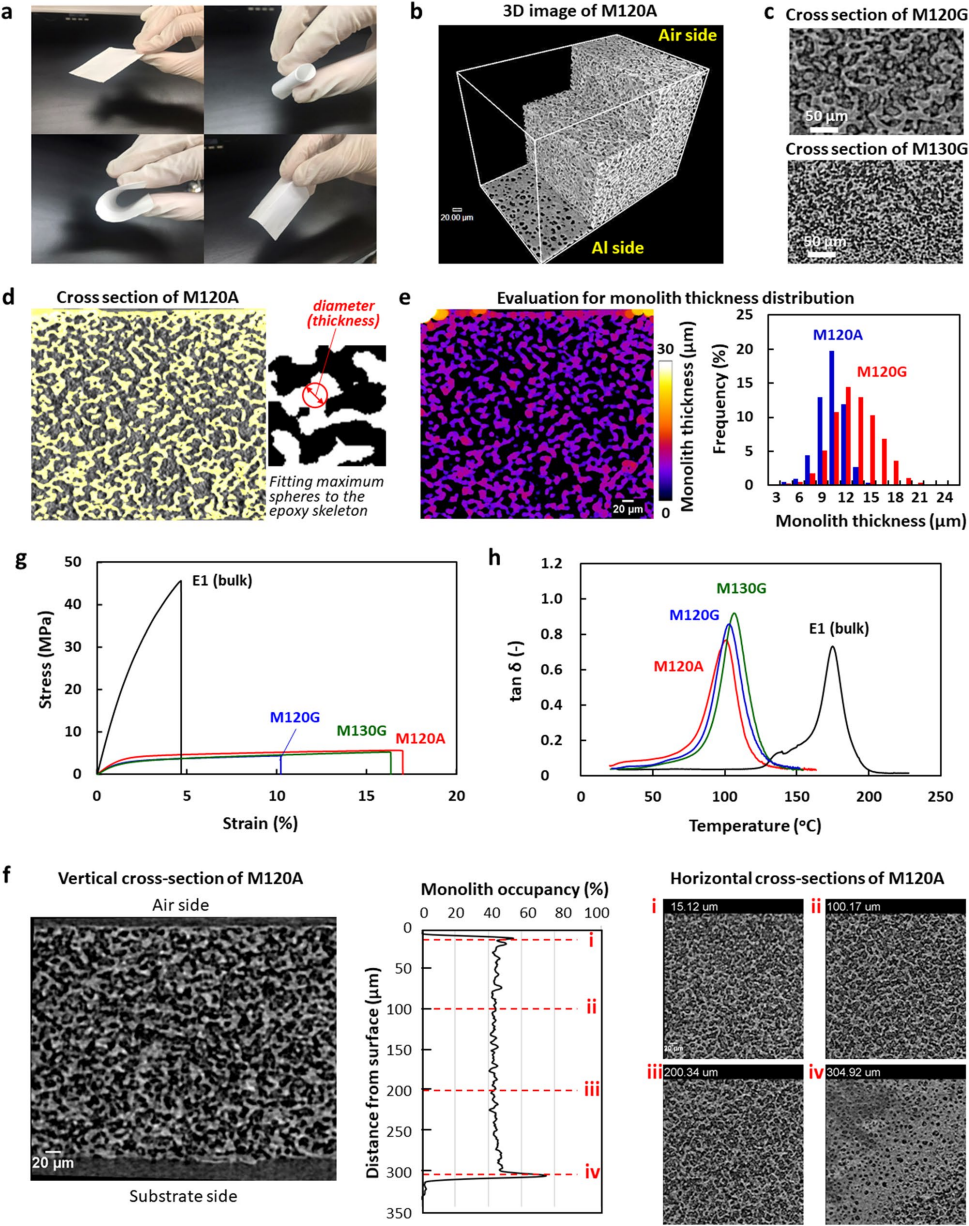
Porous structure (**a**–**f**) and mechanical and thermal properties (**g**,**h**) of the monolith sheets. (**a**) Appearance of epoxy monolith sheets. The thickness was 326 µm and 168 µm for the left and right images, respectively. The thin monolith sheet was foldable like paper. (**b**) 3D X-ray CT image of monolith sheet (M120A). (**c**) Cross-sectional images of monolith sheets with different monolith thicknesses (M120G and M130G). (**d**) Evaluation of local thickness of the epoxy skeleton for M120A, determined by fitting maximum spheres to the structure, according to the method described in the literature (ref. 45). The contribution due to the epoxy particles present in the pores was excluded during the estimation of the epoxy pillar thickness and the pore size. (**e**) Distribution of the monolith thickness for M120A (left figure). The blue and red curves in the right figure are the distribution for M120A with a fine structure and M120G with a coarse structure. (**f**) Vertical (left) and horizontal (right) cross-section images of M120A and the monolith occupancy as the function of the distance from the air-side surface (center). (**g**,**h**) Stress–strain curves of the tensile test and tanδ peaks from the DMA measurements for the monolith sheets with different coarseness (M120G, M120A, and M130G) and the bulk thermoset of E1.

A mixture of an epoxy resin (E1), a diamine as the curing agent (BACM), and a porogen (PEG200) shown in Fig. 1a was thermally cured on a glass or aluminum (Al) plate in order to fabricate the epoxy monolith (Fig. 1c). After immersion in water to remove the porogen, a flexible and foldable monolith sheet, as shown in Fig. 2a, was peeled from the plate. This unique flexible property is due to the presence of continuous pores in the sheet. Non-destructive observations using a 3D X-ray imaging technique^38,39^ can visualize the porous structure of the epoxy monolith. In this imaging method, any desired 3D and cross-sectional (2D) images are visualized by computer tomography from X-ray projection images for various kinds of composites^40–44^. Figure 2b shows the 3D structures of an exfoliated monolith sheet. The 3D X-ray imaging has revealed the fine structure of the internal epoxy skeleton. In this study, several monolith sheets with different sizes of the porous structures were prepared by changing the fabrication conditions, such as the curing temperature (120 or 130 °C) and the substrate (a glass or Al plate). As shown in the cross-sectional images of the X-ray CT in Fig. 2c, the sizes of the epoxy frames and pores were variable depending on the curing conditions of the epoxy. In Fig. 2d, the monolith skeletons are emphasized by a beige color. The average thickness of the monolith skeleton can be assessed using the local thickness determined by fitting maximum spheres to the structure^45^. Figure 2e illustrates the distribution of the monolith thickness for the inner structure of the sheets. The size of the continuous epoxy skeleton significantly depended on the conditions for the monolith fabrication; i.e., the epoxy monolith sheets consisting of fine pillars with a 7.5 ± 1.8 µm thickness during the fabrication using the Al plate with a high thermal conductivity, while the monolith with a larger thickness and broad distribution (10.3 ± 2.8 µm) was produced on the glass plate. As the curing temperature increased, the pillar diameter decreased (Fig. 2c). It was previously reported that the monolith structure also depended on the formulation of the epoxy resins (See Supplementary Table 1 in Supporting Information)^35^. A fine monolith structure was formed by the fast cross-linking reaction at an earlier stage during the Ostwald propagation of the phase-separated structure via a spinodal decomposition^30^. Figure 2f indicates the epoxy occupancy as a function of the position along a direction vertical to the sheet plane. It revealed the presence of skin layers with a thickness less than 10 μm in the areas near both surfaces. An epoxy-rich phase is favorably distributed at the surface (closed to air) and the interface (closed to the substrate) with a higher free energy during the monolith fabrication process^34^.

**Table 1:** Thermal and mechanical properties of the monolith sheets, the CNPs with E2 and E3, and the bulk thermosets of E1 and E2.

Material	Sample code	*T*_g_^a^ (^o^C)	*E*’ at 25 ^o^C^a^ (MPa)	Strength at break^b^ (MPa)	Strain at break^b^ (%)	Modulus^b^ (MPa)
Monolith sheet^c^	M120G	103	319	5.03 ± 0.63	10.7 ± 0.5	318 ± 37
	M120A	102	521	6.26 ± 0.57	16.2 ± 2.1	394 ± 24
	M130G	107(100)	450	5.55 ± 0.21	17.2 ± 1.2	290 ± 14
Co-continuous network polymer^d^	CNP-E2	‒ 0.3, 78.5	266	8.31 ± 0.67 (0.67 ± 0.14)^e^	11.4 ± 1.5 (17.4 ± 6.8)^e^	253 ± 35 (4.9 ± 0.7)^e^
	CNP-E3	‒ 38.2, 20.4	34	1.84 ± 0.58	41.5 ± 14.3	11.9 ± 0.8
Bulk thermoset^r^	E1	175	1950	45.8 ± 6.1	4.6 ± 1.1	1640 ± 71
	E2	‒	‒	0.70 ± 0.02	31.5 ± 0.9	3.5 ± 0.2

^a^Based on tanδ peak temperature of DMA measured at the frequency of 1 Hz and the heating rate of 2 °C/min. Values in parentheses indicate *T*_g_ values determined by DSC from the second heating process at the heating rate of 10 °C/min.

^b^Based on the tensile test at the rate of 1 mm/min and room temperature. Modulus was determined in the strain range of 0.05–0.25%

^c^M120G and M120A were prepared on glass and Al plates, respectively, at 120 °C. M130G was prepared on a glass plate at 130 °C.

^d^CNP-E2 and CNP-E3 were prepared using M120A as the monolith sheet.

^e^Values in parentheses indicate those determined at 80 °C.

^f^Bulk thermoset prepared by thermal curing of an epoxy resin (E1 or E2) with BACM in the absence of PEG200 as the porogen.

The thermal and mechanical properties of the monolith sheets are summarized in Table 1. There is no doubt that the glass transition temperature (*T*_g_) and the storage modulus (*E’*) of the monolith sheets are significantly low compared to those of the corresponding bulk thermoset made in the absence of the porogen (Fig. 2g,h). The *T*_g_ and *E’* values estimated by DMA were 102–107 °C and 319–521 MPa (at 25 °C) for the monolith sheets, respectively. The *T*_g_ and *E’* values of E1 as the bulk thermoset were 175 °C and 1950 MPa, respectively. The strength at break of the monoliths was as low as 5.3–6.3 MPa for the tensile test, being independent of the coarse structure of the monoliths. These strength values were one tenth of the value for the bulk thermoset (45.8 MPa). The difference in the mechanical properties of the monolith and bulk materials is related to the presence or absence of a porous structure. On the other hand, the thermal property of the monolith (Fig. 2h) cannot be explained by the porous structure. It is assumed that the PEG200 incorporated inside the epoxy cure remains and acts as a plasticizer during the fabrication of the monolith^30^.

Thus, the major factor why the monolith sheets exhibit mechanical properties different from the bulk cured product is ascribed to the porous structure inside the monolith. Therefore, we have proposed a toughening of the epoxy monolith by filling the pores with different flexible and stretchable cured materials, which are expected to compensate for the disadvantages of the hard and brittle epoxy resins.

## CNP filled with epoxy resins

**Figure 3:**
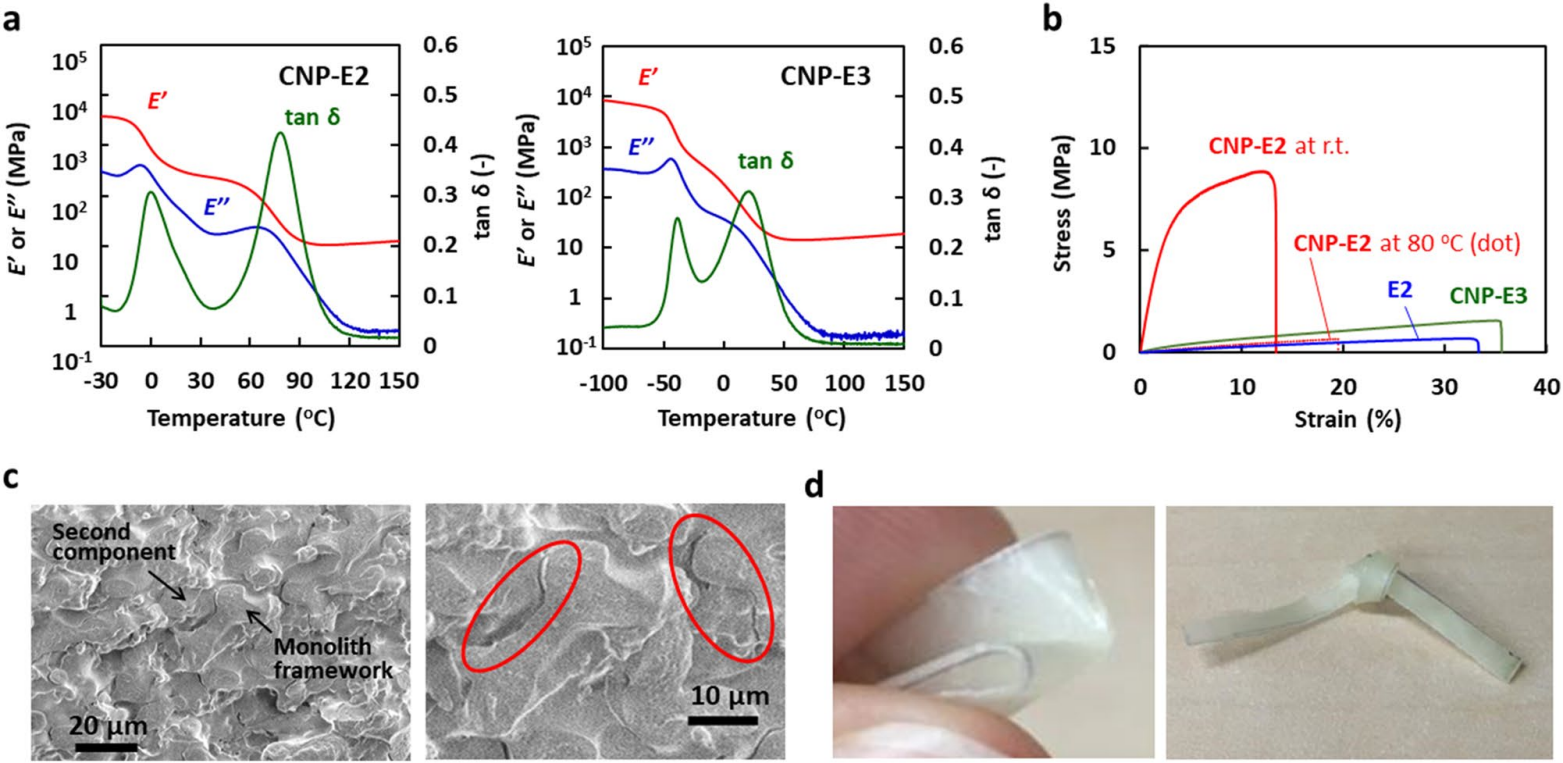
Thermal and mechanical properties of the CNPs filled with soft epoxy resin. (**a**) Temperature dependence of *E’* and tanδ for CNP-E2 and CNP-E3 determined by DMA at a frequency of 1 Hz and the heating rate of 2 °C/min. (**b**) Stress–strain curves of CNP-E2 measured at room temperature and 80 °C, CNP-E3 at room temperature, and the bulk thermoset of E2 at room temperature. (**c**) SEM image of the fracture surface of CNP-E2 after the tensile test. Some gaps are detected at the interface of the two components. The right image is expanded and red ellipsoids indicate the formation of large gaps between the first and second components. In these SEM images, each co-continuous domain of the two different epoxies is distinctly observed. Smaller pores with a size less than a micrometer are seen in the monolith skeleton, being formed by a secondary phase separation during the first fabrication process of the epoxy monolith (see also ref. 30). (**d**) Photographs of the folded and tied CNP-E2. For the fabrication of the CNPs filled with epoxy resins, M120A was used as the monolith sheet.

The low-*T*_g_ epoxy resins (Fig. 1b) were initially used as the second component to fabricate the epoxy/epoxy CNPs, in which two kinds of epoxy resins with different characteristics have independent continua. The CNPs were semi-transparent materials, being in contrast to the opaque monolith sheet that strongly scatters visible light. The SEM image of the CNP cross-section showed that the pores of the original monolith were filled with the second epoxy resin without gaps. The DMA curves of the CNP-E2 and CNP-E3 (Fig. 3a) indicate a two-step glass transition for both composites, of which the peak on the lower temperature side is attributed to the *T*_g_ of the E2 or E3 network, and the higher temperature peak is of the hard network of E1. The *T*_g_ values of the hard component are lower than those of the bulk thermoset and the monolith before filling the second soft epoxy (see also Table 1). In addition, they depended on the *T*_g_ of the second components. It is due to the occurrence of the further reaction of an added low-*T*_g_ epoxy with the monolith component at the second curing step. The interpenetration of the second epoxy changes the mechanical property of the monolith structure as the first component. The *E’* value of CNP-E2 and CNP-E3 were 266 MPa and 34 MPa at 25 °C, respectively, which are lower than 521 MPa for the original monolith. Thus, it has been clarified that the second epoxy resin penetrates into the hard epoxy skeleton during the fabrication process of the CNPs, and leads to lowering the *T*_g_ of the hard epoxy component.

Both the elastic and plastic deformation processes were confirmed in the stress–strain curve of CNP-E2 with the *T*_g_ values above and below room temperature (Fig. 3b). The strength at break reached 8.3 MPa, which was greater than the value for the monolith sheets. The shape of the curve for CNP-E2 was similar to that of the epoxy monolith without filling the second epoxy except for the breaking strength (Fig. 2g). In contrast, another composite, CNP-E3, showed a soft appearance and formed a stress–strain curve peculiar to ductile materials at room temperature. When the tensile test of CNP-E2 was conducted at a temperature higher than the *T*_g_, the curve showed elastic deformation with a small slope. This temperature-dependent mechanical property was reversible. In the SEM images of a test piece of CNP-E2 after the tensile measurement (Fig. 3c), the fracture surface was not smooth and contained many small undulations. In the expanded images, some gaps between the two components were detected but the continuous structure of each component remained. The two components were distinguishable because one of them included many small pores in the epoxy skeleton^30^. Consequently, CNP-E2 has an excellent fracture toughness. In fact, it can be folded and knotted (Fig. 3d). Interestingly, the strain at break as well as the toughness increased with an increase in the structural coarseness of the used monolith (see Supplementary Table 1 in Supporting Information), although the strength at break simultaneously decreased. Thus, it has been revealed that the mechanical strength and flexibility of the CNPs depend on the microstructure of the co-continuous components.

**Figure 4:**
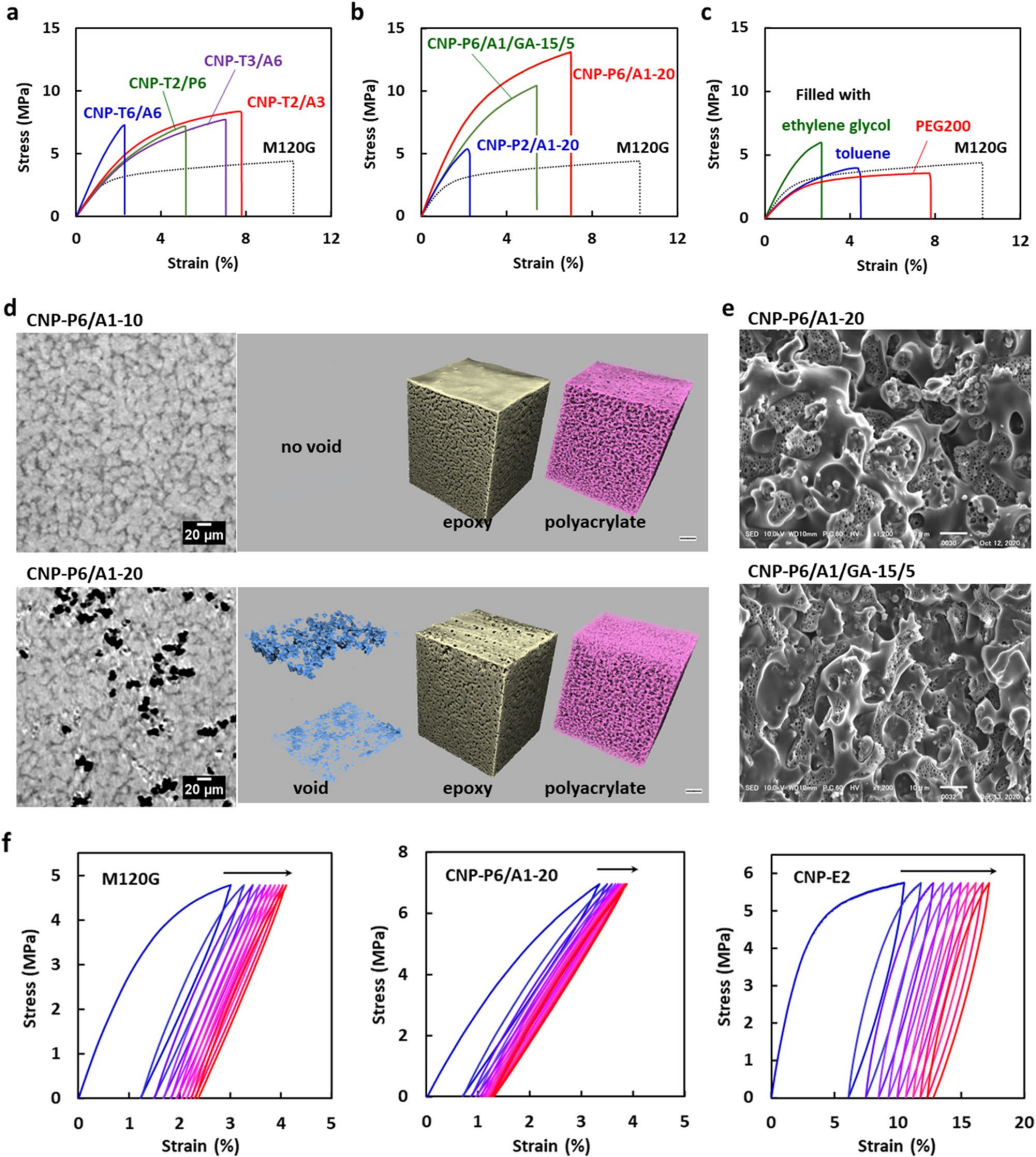
Mechanical properties and X-ray CT and SEM images of the CNPs filled with thiol–ene thermosets and polyacrylates. (**a**) Stress–strain curves of the CNPs filled with thiol–ene thermosets. See Fig. 1b for the chemical structure of the polyfunctional thiols and acrylates. (**b**) Stress–strain curves of the CNPs filled with polyacrylates. (**c**) Stress–strain curves of the monolith sheet (M120G) filled with PEG200, ethylene glycol, and toluene as the liquid materials for the second component. (**d**) X-ray CT images of CNP-P6/A1-10 and CNP-P6/A1-20. Cross-section images are reconstructed in the horizontal direction for the area near the bottom surface of the CNPs. The X-ray CT images were analyzed by the phase contrast imaging (PCI) method (ref.^50^) and they are represented for each component, i.e., the polyacrylate, the epoxy, and the void corresponding to the light gray, dark grey, and black areas in the left cross-section images. (**e**) SEM images of the fracture surfaces of CNP-P6/A1-20 and CNP-P6/A1/GA-15/5 after the tensile test. (**f**) Hysteresis stress–strain curves of the monolith sheet (M120G, left), CNP-P6/A1-20 (center), and CNP-E2 (right). The maximum applied forces were 20, 30, and 40 N, respectively.

In comparison with the mechanical properties of the bulk thermoset, it is clear that the monolith sheets exhibit a large deformation at a weaker applied force, as already shown in Fig. 2g. The deformation process of the porous materials, such as the monolith, includes the macroscopic morphological change of a mesh structure, being represented by the KIRIGAMI model^46–48^. For the deformation of the monolith, the epoxy skeleton with a high elasticity undergoes a slight bending and twisting, but they can result in a large deformation of the entire monolith with a small external stress. In an initial region for the stress‒strain relationship of the monoliths (for example, less than 1% strain for M120G), the deformation process was almost reversible, with very little hysteresis. Further stretching caused irreversible and partial breakage of the epoxy skeleton, which creased a large hysteresis (See also the results for repeated tensile tests in Fig. 4f and Supplementary Fig. 8). For the CNPs, the fracture of the hard epoxy component occurs during the large deformation, but the destruction of the entire material is suppressed due to the flexible second epoxy networks.

## CNP filled with acrylic thermosets

CNP was next prepared by thiol‒ene thermosets using polyfunctional thiols and acrylates as the secondary filling component (Fig. 1b). Similar to the CNP filled with the low-*T*_g_ epoxy, the SEM images of the cross section confirmed that the monolith pores were successfully filled with the second component without any gaps. Based on the DMA curves, the two peaks due to the expected two kinds of transitions were confirmed (Table 2). The *T*_g_ observed in the high temperature region was high and independent of the *T*_g_ of the second components. It supports the fact that the thiol‒ene reaction at the second stage orthogonally proceeds^49^ without affecting the physical properties of the first epoxy component. In Fig. 4a and Table 2, the results of a tensile test are summarized for the CNPs prepared using the various thiol‒ene thermosets. The strength at break for CNP-T6/A6 was improved to 1.7 times that of the original monolith, but the strain was reduced to 1/3. In the system with hexafunctional thiols and acrylates, the cross-linking density of the second network is too high, leading to the formation of a brittle composite. When the number of functional groups of each compound was reduced to 2 or 3 (T2, T3, or A3), the strength increased to 6.4‒7.3 MPa while maintaining the elongation property of the original monolith. In Table 2, the *T*_g_ values are also summarized for the CNPs as well as the values for the second networks. They support the enlarged toughness for the CNPs containing the second components in the rubber state. It was also clarified that a composite exhibiting high strength and strain values was available when P6, which is a star-shaped PEG with six terminal acrylate moieties (see Fig. 1b), was used in combination with T2. Therefore, the scope of the materials design was expanded to the radical polymerization system of acrylates.

**Table 2:** Thermal and mechanical properties of the CNPs using M120G as the monolith sheet and acrylic thermosets as the second component.

Sample code	*T*_g_^a^ (^o^C)	Strength at break^b^ (MPa)	Strain at break^b^ (%)	Modulus^b^ (MPa)	Toughness^c^ (kJ/m^2^)
CNP-T6/A6^d^	48 [57], 140	7.29	2.28	396	0.94
CNP-T3/A6^e^	9.8 [21], 150	6.40 ± 1.01	6.07 ± 1.01	207 ± 9.7	2.6 ± 0.84
CNP-T2/A3	[‒ 12]	7.15 ± 1.12	6.08 ± 1.71	210 ± 51	3.0 ± 1.5
CNP-T2/P6	‒ 42 [‒42]	7.25 ± 0.056	5.30 ± 0.13	219 ± 15	2.3 ± 0.04
CNP-P6f.^,g^	‒ 44, 117	8.00 ± 1.50	9.76 ± 1.94	227 ± 22	5.5 ± 2.0
CNP-P6/A1-10	‒	9.47 ± 0.97	4.18 ± 1.23	356 ± 50	2.5 ± 1.0
CNP-P6/A1-20^ h^	‒ 50 [‒ 42], 121	12.4 ± 0.67	7.12 ± 0.11	437 ± 26	6.1 ± 0.20
CNP-P6/A1-30	‒	11.3 ± 0.36	5.67 ± 0.61	444 ± 3.2	4.3 ± 0.67
CNP-P6/A1/GA-18/2	‒	7.50 ± 0.71	3.91 ± 1.20	329 ± 65	1.9 ± 0.76
CNP-P2/A1-20	[‒ 28]	4.36 ± 0.54	1.44 ± 0.00	388 ± 26	0.34 ± 0.04

^a^Based on the tanδ peak temperature of DMA measured at a frequency of 1 Hz and a heating rate of 2 °C/min. Values in bracket indicate *T*_g_ values for the bulk thermosets of the second component determined by DMA.

^b^Based on the tensile test at the rate of 1 mm/min and room temperature. The modulus was determined in the strain range of 0.05–0.25%

^c^Evaluated based on an area below each stress–strain curve. The toughness values of M120G was 4.4 ± 0.77 kJ/m^2^.

^d^ The *E’* values of CNP-T6/A6 were 1680, 309, and 8.9 MPa at 25, 100, and 180 °C, respectively, determined by DMA.

^e^The *E’* values of CNP-T3/A6 were 240 and 17.5 MPa at 80 and 180 °C, respectively, determined by DMA.

^f^M120A was used instead of M120G.

^g^The *E’* values of CNP-P6 were 3170, 324, and 23.1 MPa at ‒80, 0, and 160 °C, respectively, determined by DMA.

^h^The *E’* values of CNP-P6/A1-20 were 5530, 839, and 29.6 MPa at ‒80, 0, and 160 °C, respectively, determined by DMA.

When radical polymerization of an acrylic monomer is used for filling the pores, the problem of volume shrinkage associated with the polymerization is inevitable because it is an inherent feature of the vinyl polymerization. In fact, attempting to produce the CNPs using conventional acrylic monomers such as A1 and A6, caused a large warpage and undulation of the composites. This is unlike the negligible shrinkage that was seen in the epoxy and thiol‒ene curing systems, as already described. In contrast to the volume-shrinking conventional monomers, the single use of P6 provided a flexible CNP of which the strength and stain values at break were as high as 8.0 MPa and 9.8%, respectively. The modulus was somewhat low at 227 MPa. The cross-sectional SEM image of CNP-P6 revealed that there were no gaps at the interface between the co-continuous dual components. In the copolymerization system of P6 and A1, the gaps increased as the composition of A1 increased and many voids were detected for the samples including 20% of A1 (Fig. 4d,e). The X-ray CT imaging has confirmed that the voids are unevenly distributed in the CNPs. As the results of the mechanical tests, the CNP-P6/A1-20 exhibited the highest strength and toughness (Table 2). On the other hand, the toughness was significantly reduced for CNP-P2/A1-20 with a low cross-linking density for the second network, which was prepared using a telechelic polyacrylate P2 in place of the multifunctional P6.

In order to discuss the effect of the gaps between the two co-continuous structures on the mechanical properties, we prepared the CNPs in the presence of GA (See Fig. 1b for the chemical structure) during the thermal curing process at the second step. The glycidyl group of GA is expected to react with the functional groups in the epoxy monolith skeleton and to suppress the formation of gaps in the CNP. As the results of the X-ray CT imaging and the tensile test, it has been clarified that these is no gap in the produced CNPs and the strength and strain values at break are lower, as shown in Table 2 and Fig. 4b (See also X-ray CT images in Supplementary Fig. 7). They indicated the important role of the sliding of mutual components on resistance to mechanical fracture during the large deformation of the CNPs, leading to effective energy dissipation to prevent macroscopic material destruction. In this study, a repeated tensile test was also conducted. During the repeated measurements under the maximum applied forces of 20‒40 N, the cyclic curves shifted with a hysteresis as shown in Fig. 4f and the degree of the shift for the CNP (CNP-P6/A1-20) was smaller than that of the original monolith (M120G). This indicates the suppression of the fatigue of the rigid epoxy continuum surrounded by the flexible elastomer as the second component. The epoxy/epoxy CNPs gave similar trajectory, but the pushback effect of the second component has become weaker. Furthermore, it was revealed that the CNPs containing a viscous liquid as the second component resulted in no improvement in the strength (Fig. 4c). Similar results were obtained for the sample without removing the porogen after the curing of the first epoxy material during the fabrication process of the monolith sheets. Thus, the liquid materials act like a plasticizer for the CNP, but no toughening effect.

## Outlook

In this study, we have successfully fabricated the CNPs as new composite materials and evaluated their strength and toughness. The epoxy monolith is a unique material, which has co-continuous structure of an epoxy skeleton and pores leading to its flexible and foldable properties despite the epoxy cured materials with a high *T*_g_. The CNPs were prepared by filling the voids in the monolith with any second cross-linked polymers. Based on the results of three systems using epoxy, thiol‒ene thermosets, and polyacrylates as the second filler components, we determined the requirements for strengthening the CNP as follows. The epoxy monolith is in a glassy state and the second one should be a moderately cross-linked polymer in the rubbery state. In the case of DN gels as the most typical example of artificial toughened polymers by the sacrificial fracture approach, the structural features of the DN gels are summarized as follows^24^: The first network has a rigid structure and the second network has a flexible structure. The concentration of the second network is much higher than that of the first network, and the first network is densely cross-linked. When the second network is loosely or not cross-linked, the gels become stronger. The sacrificial fracture mechanism has been applied to soft materials, such as DN gel and elastomers. In this study, we have demonstrated that the sacrificial fracture mechanism is adjustable for toughening the CNPs as the hard composites as well as the unique mechanical property of the epoxy monolith itself. Some features for the fracture of the toughened epoxy composites may be different from those of the soft materials. The monolith structure can be fabricated using various materials other than epoxy resin. Therefore, the variation in the combination of the dual network polymer materials of the high-strength CNPs will be expanded in the future, leading to their use in a wide range of application fields.

## Supplementary Information

Supplementary Video 1.

Supplementary Video 2.

Supplementary Video 3.

Supplementary Video 4.

Supplementary Video 5.

Supplementary Information.
