# Supplementary material for: Co-continuous network polymers using epoxy monolith for the design of tough materials
Source: Sci Rep. 2021 Jan 14;11:1431. doi: 10.1038/s41598-021-80978-2 (PMC7809133; doi:10.1038/s41598-021-80978-2)
Supplement: Supplementary file 6 — Supplementary Information. [file 41598_2021_80978_MOESM6_ESM.pdf]

Supplementary Information

Co-continuous Network Polymers Using Epoxy Monolith for the Design of Tough Materials

Ren Tominaga,[1] Yukihiro Nishimura,[1] Yasuhito Suzuki,[1] Yoshihiro Takeda,[2] Masaru Kotera,[3] and Akikazu Matsumoto[1,*]

[1]*Department of Applied Chemistry, Graduate School of Engineering, Osaka Prefecture University, 1-1, Gakuen-cho, Naka-ku, Sakai, Osaka 599-8531, Japan*
[2] *Core Technology Research Department, X-ray Research Laboratory, Rigaku Corporation, 3-9-12, Matsubara-cho, Akishima, Tokyo 196-8666, Japan*
[3] *R&D Department, Hotmelt Adhesive Division, MORESCO Corporation, 5-5-3, Minatojimaminami-machi, Chuo-ku, Kobe, Hyogo 650-0047, Japan.*
** Correspondence to Akikazu Matsumoto, Tel: +81-72-254-9292, Fax: +81-72-254-9292,*
E-mail: matsumoto@chem.osakafu-u.ac.jp

Contents

Experimental Methods. General Procedure, X-ray Imaging, Materials, Monolith Fabrication, and Synthesis of the CNPs

Supplementary Table 1. Mechanical properties of the **CNP**s fabricated using monolith sheets with different pore sizes prepared under various conditions.

Supplementary Table 2. Physical properties of the bulk thermoset materials as the second components used for the fabrication of the thiol–ene and polyacrylate **CNP**s.

Supplementary Table 3. Mechanical properties of the **CNP**s using **M120G** and **P6/A1/GA** as the monolith and second component, respectively.

Supplementary Figure 1. Time–temperature curves during the fabrication process of monolith sheets.

Supplementary Figure 2. DMA curves of the monolith sheets at the frequency of 1 Hz and the heating rate of 2 ^{o}C/min.

Supplementary Figure 3. DSC and DMA curves for the **CNP**s and the thiol–ene thermosets.

Supplementary Figure 4. DMA curves for the **CNP**s filled with the polyacrylates.

Supplementary Figure 5. SEM images of the fracture surfaces for **CNP-E3**, **CNP-E2**, **CNP-T3/A6**, and **CNP-T6/A6** after the tensile test.

Supplementary Figure 6. SEM images of the fracture surfaces for **CNP-P6/A1**s and **CNP-P6/A1/GA-15/5**.

Supplementary Figure 7. X-ray CT images of the **CNP**s (reconstructed cross sections).

Supplementary Figure 8. Hysteresis stress–strain curve of the **CNP**s at an initial stage of expansion.

Movies of X-ray CT images for M120G, M120A, CNP-P6/A1-20, and CNP-P6/A1/GA-18/2.

Experimental Methods

General Procedure. The SEM observation was performed using VE-9800 (Keyence Corporation, Ltd., Osaka, Japan) with an acceleration voltage of 1.0 kV and Au vapor deposition or JSM-IT100 (JEOL, Ltd., Tokyo, Japan) with an acceleration voltage of 10 kV and Os vapor deposition. The differential scanning calorimetry (DSC) measurement was carried out using DSC-60 (Shimadzu Corporation, Kyoto, Japan) at the heating rate of 10 °C/min in a nitrogen stream. The T_g was determined from a trace during the second heating process. DMA was carried out using similar test pieces and DMS 6100 (Seiko Instruments, Inc., Tokyo, Japan). The conditions were a dual cantilever mode at the heating rate of 2 °C/min. Sinusoidal strains with an amplitude of 10 µm at 1 Hz were applied. The T_g was determined as the peak temperature of the tanδ curves. The tensile test was carried out and the tensile rate of 1.0 mm/min using an Autograph AGSX 5 kN (Shimadzu Corporation, Ltd., Kyoto, Japan) at room temperature. The size of the test pieces was 10 mm × 40 mm. The thickness was determined using a Peacock dial thickness gauge (Ozaki Mfg. Corporation, Ltd., Osaka, Japan). The sample number (N) was 2−5 for the tensile measurements except for the single measurement for **CNP-T6/A6** in Table 2. The modulus was determined from the initial slope of the stress−strain curve in the range of elongation of 0.05%−0.25%. The cycle number was 5−10 for the cyclic tensile test.

X-ray Imaging. The 3D X-ray imaging was carried out using an X-ray microscope CT nano3DX, Rigaku Corporation, Tokyo, Japan. The sample was placed on a 2-axis goniometer stage. A Cu target (K_α, λ = 0.15418 nm, 8.048 keV) was chosen to visualize the fine structure of the epoxy monoliths. The tube voltage and current were set to 40 kV and 30 mA, respectively. The field of view (FOV) for the camera was 0.66 mm x 0.66 mm and effective pixel was 0.62 µm. The goniometer stage was rotated 180 degrees and 600 projection images were taken. The scan time was 15−60 min. The FDK (Feldkamp–Davis–Kress) algorithm was then used to reconstruct the tomograms.

Materials. 2,2'-Bis(4'-glycidyloxaphenyl)propane (**E1**) as the epoxy resin, 4,4'-methylenebis-(cyclohexylamine) (BACM) as the curing agent, poly(ethylene glycol) (PEG200, M_n = 200) as the porogen were purchased from Tokyo Chemical Industry Corporation, Ltd., Tokyo, Japan, and used as received. M_n is the number-average molecular weight. Tripropylene glycol diglycidyl ether (**E2**) and polyethylene glycol diglycidyl ether (**E3**) were commercially available as SR-TPG and SR-8EGS from Sakamoto Yakuhin Kogyo Corporation Ltd., Osaka, Japan, and used without further purification. Ethylene glycol bis(3-mercaptopropionate) (**T2**), trimethylol-propane tris(3-mercaptopropionate) (**T3**), dipentaerythritol hexakis(3-mercaptopropionate) (**T6**), *n*-butyl acrylate (**A1**), glycidyl acrylate (**GA**), and trimethylolpropane triacrylate (**A3**) were purchased from Tokyo Chemical Industry

Corporation, Ltd., Tokyo, Japan, and used as received. Dipentaerythritol hexaacrylate (**A6**) was provided from Shin-Nakamura Chemical Corporation, Ltd., Wakayama, Japan. Poly(ethylene glycol) diacrylate (**P2**, *n* = 9) was purchased from Tokyo Chemical Industry Corporation, Ltd., Tokyo, Japan, and used as received. **P6** was commercially available as SA-TE60 (the number of functional groups, 6; the number-average molecular weight, 3400) from Sakamoto Yakuhin Kogyo Corporation, Ltd., Osaka, Japan, and used without further purification. Benzoyl peroxide (BPO) as a radical initiator was purchased from Nacalai Tesque, Inc., Kyoto, Japan, and used as received. The Pyrex glass and Al plates were purchased from the AsOne Corporation, Osaka, Japan, and used after cleaning with acetone.

Monolith Fabrication. An epoxy monolith was fabricated as follows. **E1**, BACM, and PEG200 (typically 70 wt% for PEG200) at the ratio of 2[NH_2]/[epoxy] (γ value) = 1.0 were well mixed for 10 min using a planetary centrifugal mixer (AR-100, Thinky Corporation, Tokyo, Japan). The mixed paste was spread on the glass or Al plate to the desired thickness followed by thermal curing using an oven at 120 or 130 °C (VOC-210SD, Tokyo Rikakikai (EYELA) Corporation, Ltd., Tokyo, Japan) for 60–90 min. The temperature measurements of the reaction samples and the glass and Al plates were conducted using a multi-channel USB data logger (TC-08, Pico Technology, Cambridgeshire, U.K.) with a K-type thermocouple. The thermocouple was fixed with Kapton tape. After curing, the samples were washed with ion-exchanged water by ultrasonics for 5 min to remove the PEG200, stored in ion-exchanged water overnight, then dried in vacuo for 2 h at room temperature.

Synthesis of the CNPs. To the pores of the monolith sheets, an epoxy resin (**E2** or **E3**) and a diamine curing agent (BACM) at the ratio of 2[NH_2]/[epoxy] (γ value) = 1.0 were penetrated under reduced pressure, then heated in an oven at 120 °C for 60 min to obtain the epoxy/epoxy-type **CNP**s. Similarly, the thiol–ene **CNP**s were prepared at 160 °C for 2 h using a mixture of polyfunctional thiol and acrylate. For the synthesis of the polyacrylate **CNP**s, acrylate monomers penetrated into the pores of the monolith were polymerized in the presence of BPO as the radical initiator at 90 °C for 60 min. The residual free monomer was less than 2% after curing of the polyacrylate systems based on the results of an extraction experiment.

Supplementary Table 1. Mechanical properties of the **CNP**s fabricated using monolith sheets with different pore sizes prepared under various γ conditions

Conditions for CNP fabrication[a]		Strength at break[b] (MPa)	Strain at break[b] (%)	Modulus[b] (MPa)	Toughness[c] (kJ/m²)
γ[a]	average pore size at surface (μm)				
1.0	12.0 ± 1.9	8.31 ± 0.67	11.4 ± 1.5	253 ± 35	8.6 ± 1.7
1.2	17.6 ± 2.4	7.80 ± 0.99	22.8 ± 5.8	259 ± 49	15.7 ± 5.6
1.4	34.9 ± 5.2	5.90 ± 0.34	34.3 ± 7.1	210 ± 6	17.5 ± 4.2

[a] γ = 2[NH_2]/[epoxy]. See the previous report for the fabrication of the monoliths: Sugimoto, Y.; Nishimura, Y.; Uehara, F.; Matsumoto, A. *ACS Omega* **2018**, *3*, 7532-7541. [b] Based on the tensile test at the rate of 1 mm/min and room temperature. [c] Evaluated based on an area below each stress-strain curve. The toughness value of the corresponding monoliths was not measured.

Supplementary Table 2. Physical properties of the bulk thermoset materials as the second components used for the fabrication of the thiol–ene and polyacrylate **CNP**s

Bulk thermosets	T_g[a] (°C)	E' at 25 °C[a] (MPa)	E' at rubbery plateau[a] (MPa)	Cross-linking density,[b] n (mmol/cm³)
**T6/A6**	57.4 (33.2)	1865	124 (at 130 °C)	12.4
**T3/A6**	20.6 (−3.5)	146	43.5 (at 60 °C)	5.24
**T2/A3**	−12.2 (−30.8)	14.2	14.1 (at 20 °C)	1.93
**T2/P6**	−41.8 (−46.5)	–	7.84 (at −20 °C)	1.24
**T3/P6**	−39.4 (−45.4)	–	8.88 (at −20 °C)	1.41
**P6/A1-20**	−41.9 (−40.1)	21.1	21.1 (at −20 °C)	3.35

[a] Based on tanδ peak temperature of DMA measured at the frequency of 1 Hz and the heating rate of 2 °C/min. The values in parentheses indicate the T_g values determined by DSC from the second heating process at the heating rate of 10 °C/min. [b] The crosslinking density (n) was calculated using the following equation: n (mol/cm³) = $E'/(2(1+\mu)RT$. E' is the elasticity (in Pa), R is the gas constant, T is the absolute temperature, and μ is the Poisson ratio, which was assumed to be 0.5.

Supplementary Table 3. Mechanical properties of the **CNP**s using **M120G** and **P6/A1/GA** as the monolith and second component, respectively[a]

Sample code	Second component (weight ratio)	Strength at break (MPa)	Strain at break (%)	Modulus (MPa)
**CNP-P6/A1/GA-18/2**	**P6/A1/GA** = 80/18/2	7.50 ± 0.71	3.91 ± 1.20	584 ± 47<br>329 ± 65
**CNP-P6/A1/GA-15/5**	**P6/A1/GA** = 80/15/5	6.85 ± 1.72	3.64 ± 1.88	534 ± 63<br>325 ± 67

[a] Based on the tensile test at the rate of 1 mm/min and room temperature. The modulus was determined in the strain range of 0.05%–0.25%.

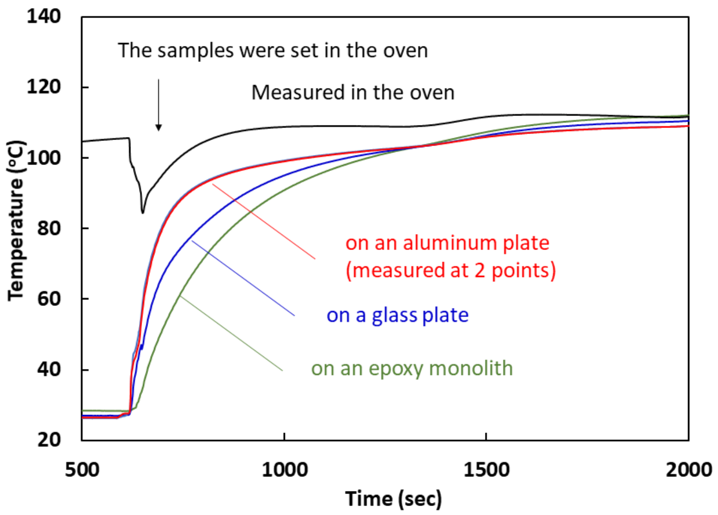

Supplementary Figure 1. Time–temperature curves during the fabrication process of monolith sheets.

The temperature profiles of the sample (epoxy monolith) and the glass and aluminum plates in an oven at 120 °C (An actual temperature was kept at 105 °C) during the fabrication process of monolith sheets.

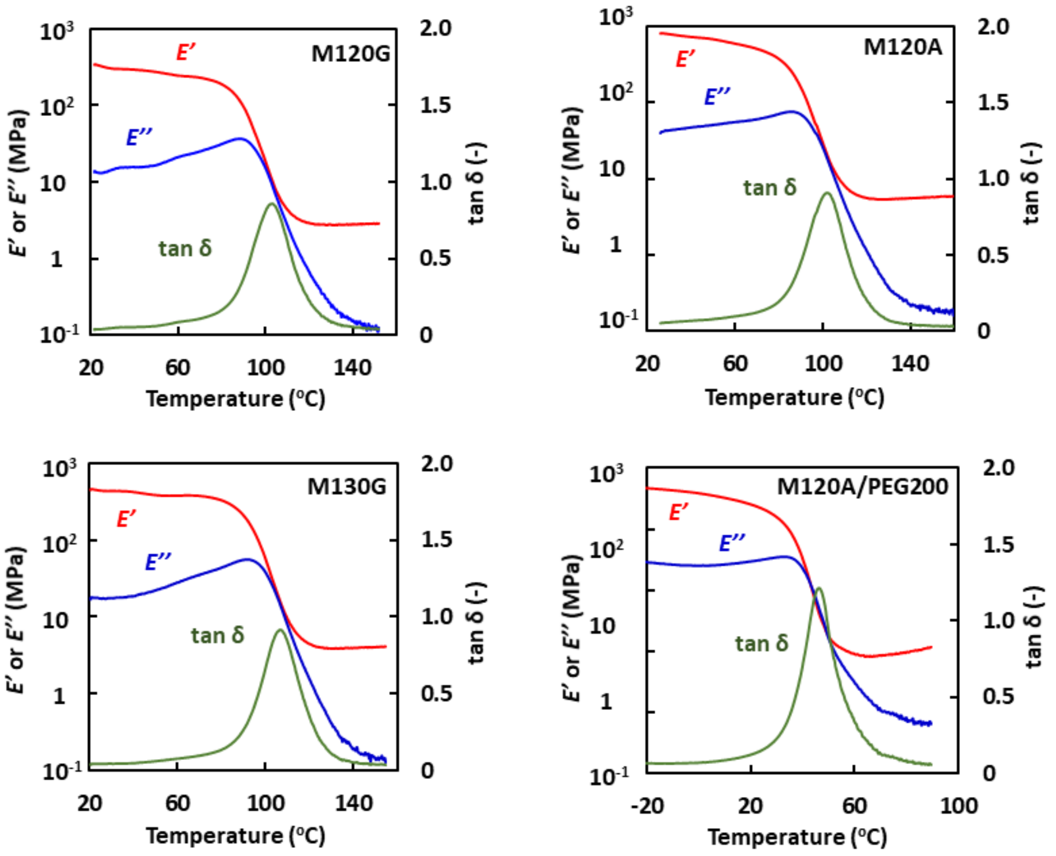

Supplementary Figure 2. DMA curves of monolith sheets at the frequency of 1 Hz and the heating rate of 2 °C/min.

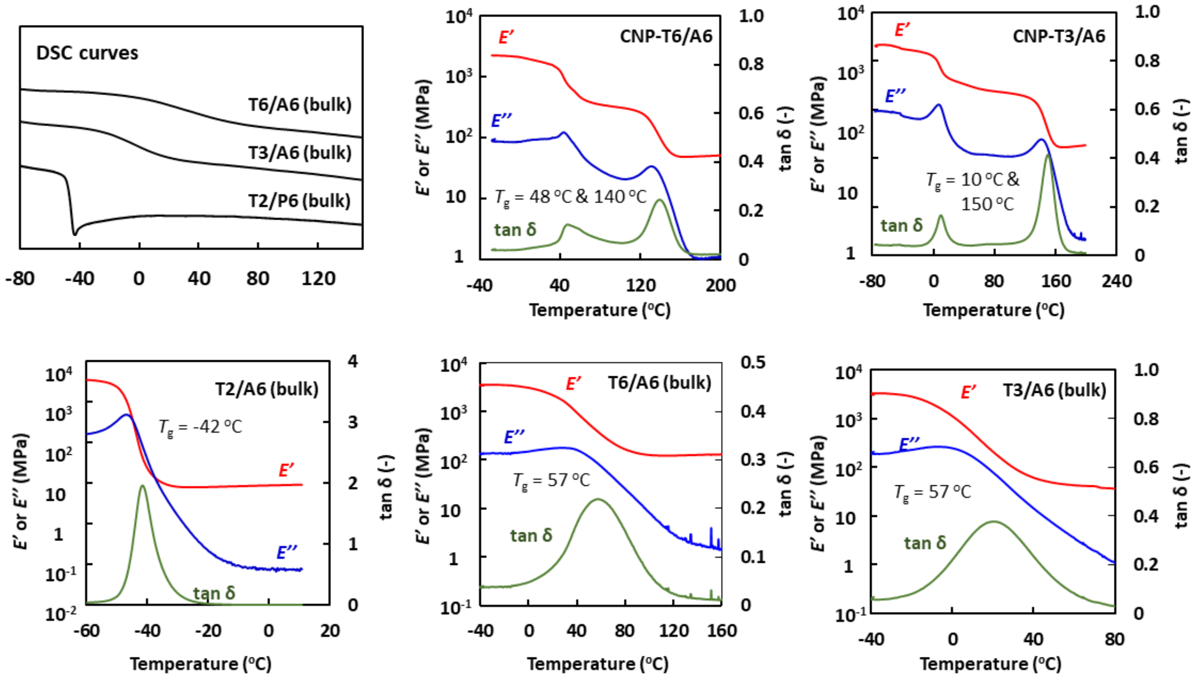

Supplementary Figure 3. DSC and DMA curves for the CNPs and the thiol–ene thermosets.

DMA was carried out at the frequency of 1 Hz and the heating rate of 2 °C/min. DSC was carried out at the heating rate of 10 °C/min in a nitrogen stream.

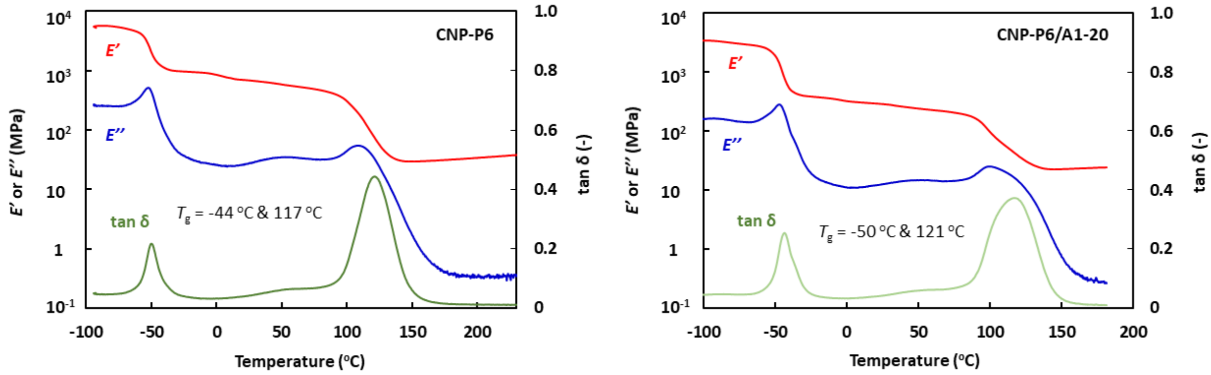

Supplementary Figure 4. DMA curves for the CNPs filled with the polyacrylates.

DMA was carried out at the frequency of 1 Hz and the heating rate of 2 °C/min.

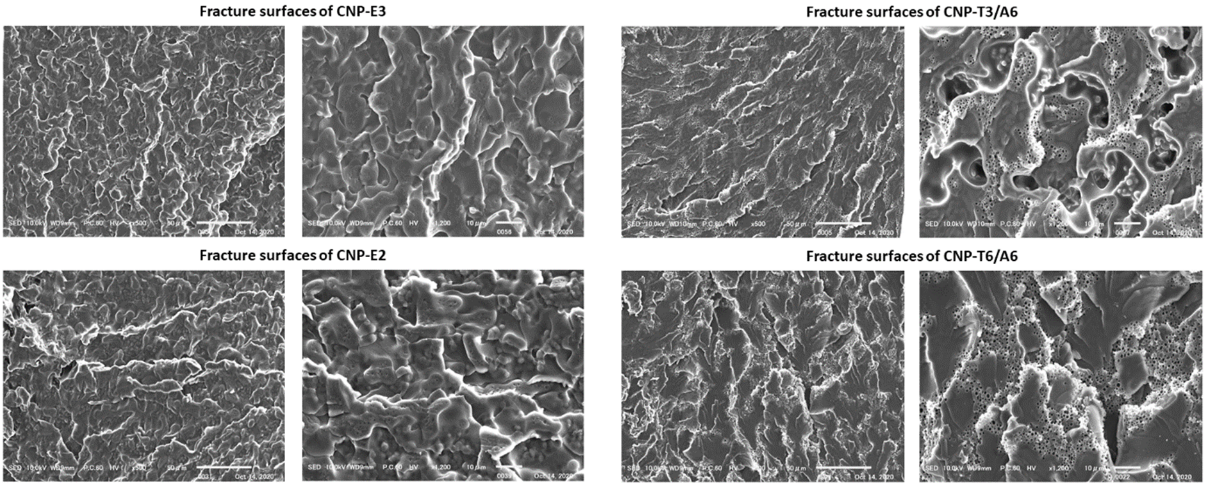

Supplementary Figure 5. SEM images of the fracture surfaces for CNP-E3, CNP-E2, CNP-T3/A6, and CNP-T6/A6 after the tensile test.

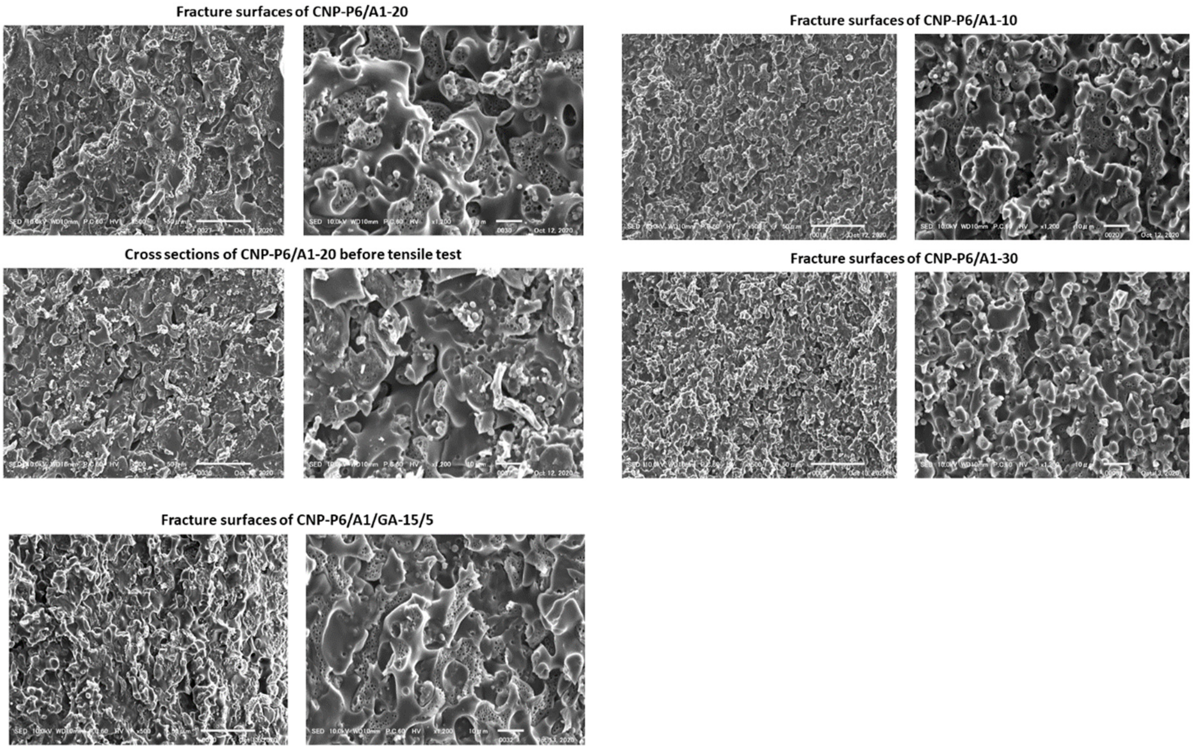

Supplementary Figure 6. SEM images of the fracture surfaces for CNP-P6/A1s and CNP-P6/A1/GA-15/5 after the tensile test. Cross sections of **CNP-P6/A1-20** before the tensile test are also shown.

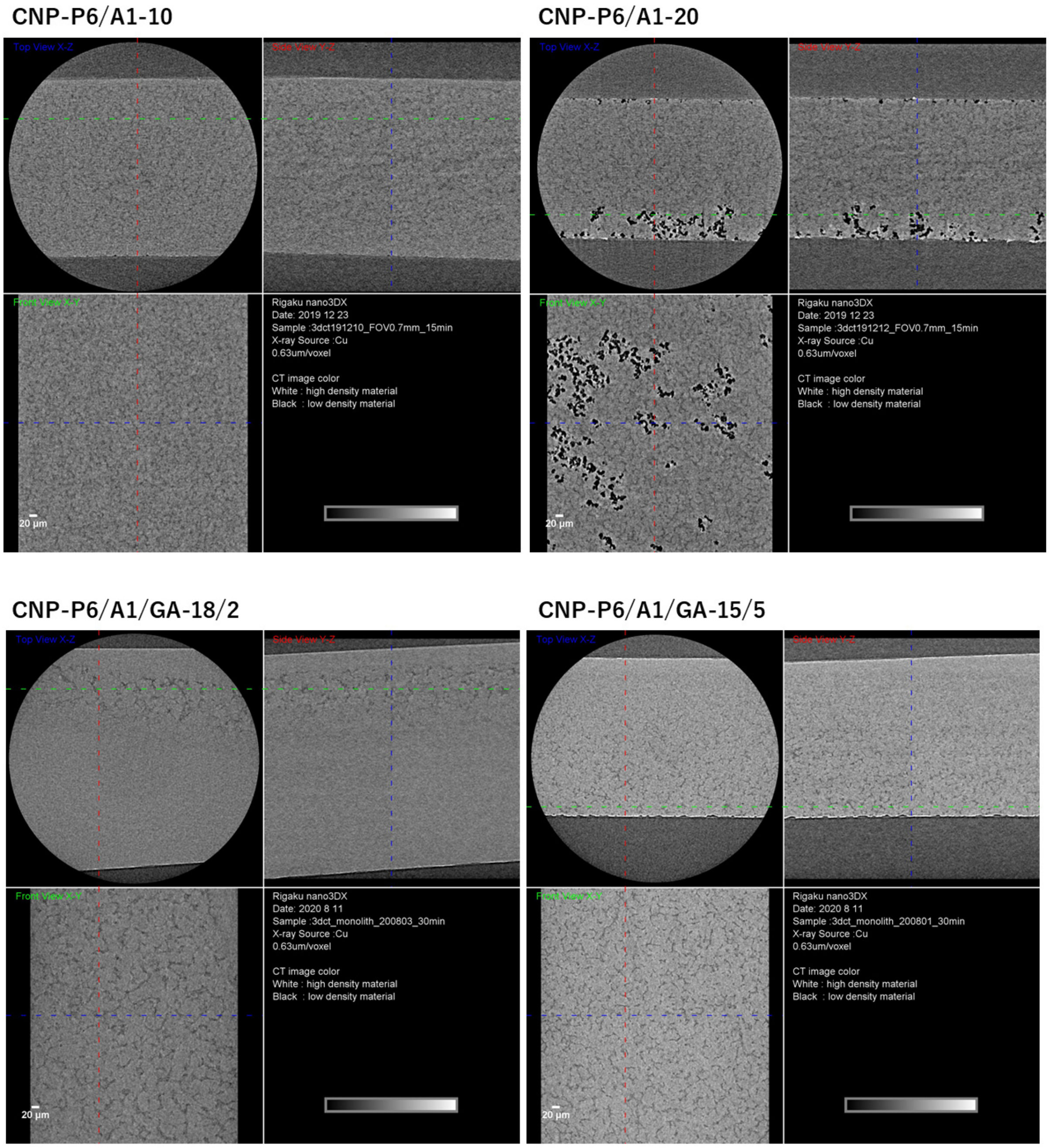

Supplementary Figure 7. X-ray CT images of the CNPs (reconstructed cross sections).

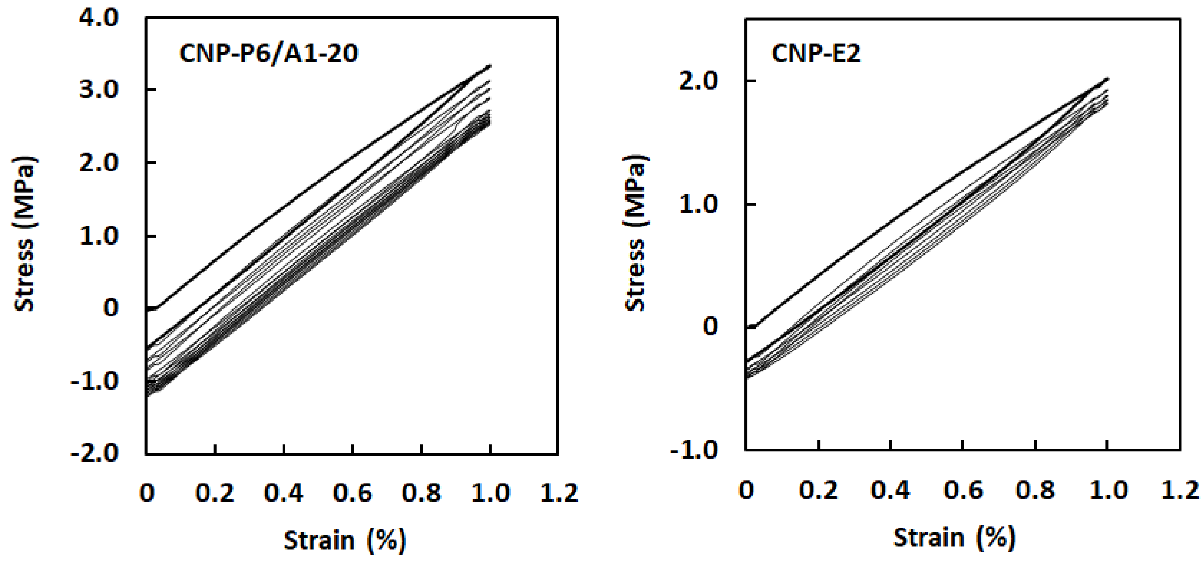

Supplementary Figure 8. Hysteresis stress–strain curve of the CNPs at an initial stage of expansion. CNP-P6/A1-20 (0–1% elongation, 10 cycles) and **CNP-E2** (0–1% elongation, 5 cycles)

Movies of X-ray CT images for M120G, M120A, CNP-P6/A1-20, and CNP-P6/A1/GA-18/2.
